# MicroRNA-361: A Multifaceted Player Regulating Tumor Aggressiveness and Tumor Microenvironment Formation

**DOI:** 10.3390/cancers11081130

**Published:** 2019-08-07

**Authors:** Daozhi Xu, Peixin Dong, Ying Xiong, Junming Yue, Kei Ihira, Yosuke Konno, Noriko Kobayashi, Yukiharu Todo, Hidemichi Watari

**Affiliations:** 1 Department of Obstetrics and Gynecology, Hokkaido University School of Medicine, Hokkaido University, Sapporo 060-8638, Japan; 2 Department of Gynecology, State Key Laboratory of Oncology in South China, Sun Yat-sen University Cancer Center, Guangzhou 510060, China; 3 Department of Pathology and Laboratory Medicine, University of Tennessee Health Science Center, Memphis, TN 38163, USA; 4 Center for Cancer Research, University of Tennessee Health Science Center, Memphis, TN 38163, USA; 5 Division of Gynecologic Oncology, National Hospital Organization, Hokkaido Cancer Center, Sapporo 003-0804, Japan

**Keywords:** microRNA-361, EMT, angiogenesis, tumor microenvironment, cancer diagnosis, cancer treatment

## Abstract

MicroRNA-361-5p (miR-361) expression frequently decreases or is lost in different types of cancers, and contributes to tumor suppression by repressing the expression of its target genes implicated in tumor growth, epithelial-to-mesenchymal transition (EMT), metastasis, drug resistance, glycolysis, angiogenesis, and inflammation. Here, we review the expression pattern of miR-361 in human tumors, describe the mechanisms responsible for its dysregulation, and discuss how miR-361 modulates the aggressive properties of tumor cells and alter the tumor microenvironment by acting as a novel tumor suppressor. Furthermore, we describe its potentials as a promising diagnostic or prognostic biomarker for cancers and a promising target for therapeutic development.

## 1. Introduction

Large-scale transcriptional analysis reveals that more than 80% of the human genome is transcribed into RNA, whereas less than 2% of the human genome is used for protein translation [1], suggesting that the vast majority of the human transcriptome is composed of non-coding RNAs (ncRNAs). MicroRNAs (miRNAs) are a class of endogenous regulatory noncoding RNAs, typically 20–23 nucleotides in length, thereby exerting essential roles in a wide range of physiological processes [2]. Although some miRNAs can bind to the 5′-untranslated regions (5′-UTRs) or the coding regions of target messenger RNAs (mRNAs) [3,4], miRNAs primarily suppress the expression of their target genes by targeting the 3′-UTRs of target mRNAs for mRNA degradation or translation inhibition [2]. A single miRNA can target many genes, and multiple miRNAs can regulate a single gene. Previous studies indicated that miRNAs may regulate as many as one third of human genes [2]. miRNAs are evolutionary highly conserved ncRNAs and are expressed in a tissue-specific or developmental stage-specific manner, thereby contributing to cell or tissue-specific protein expression profiles [5,6,7].

In human cancer cells, miRNAs exert either pro- or anti-tumorigenic effects through tissue-dependent mechanisms. Some miRNAs that are amplified or overexpressed in cancer could act as oncogenes to either directly or indirectly downregulate the expression of tumor suppressors [8,9]. On the other hand, some miRNAs, such as miR-361-5p (miR-361), can target mRNAs encoding oncogenic proteins and therefore be categorized as tumor suppressors [8,9,10].

Dysregulation of miRNA expression was reported in most cancer types [8,9]. miRNA expression profiles can distinguish cancer tissues from normal tissues and separate different cancers subtypes [11]. miRNAs have already been described as non-invasive biomarkers useful for cancer diagnosis, patient stratification, and the prediction of patient prognosis, and treatment efficacy [12,13,14].

miRNAs mediate tumor initiation and progression by regulating a variety of biological processes, including cell proliferation, migration, invasion, metastasis, glycolysis, apoptosis, cancer stem cell (CSC)-like phenotype, chemoresistance, and epithelial-to-mesenchymal transition (EMT) [11,15]. The components of the tumor microenvironment, which includes the extracellular matrix (ECM), fibroblasts, immune cells, inflammatory cells, endothelial cells, lymphatic endothelial cells, growth factors, and cytokines, play an important role during tumor progression and metastasis [16]. Recent works demonstrated the importance of miRNAs in regulating complex signaling networks involved in multiple aspects of the microenvironment remodeling, including the hypoxic response, angiogenesis, anti-tumor immune response, inflammation, and ECM organization [16].

In this review, we discuss the expression pattern of miR-361 in human tumors and the mechanisms responsible for its dysregulation. Furthermore, we elucidate the diverse mechanisms by which downregulation of miR-361 expression confers the aggressive properties of tumor cells and alters the tumor microenvironment. Finally, we describe its potential as a promising biomarker for cancer diagnosis and prediction of prognosis in patients with cancers.

## 2. Evidence Acquisition

**Figure 1:**
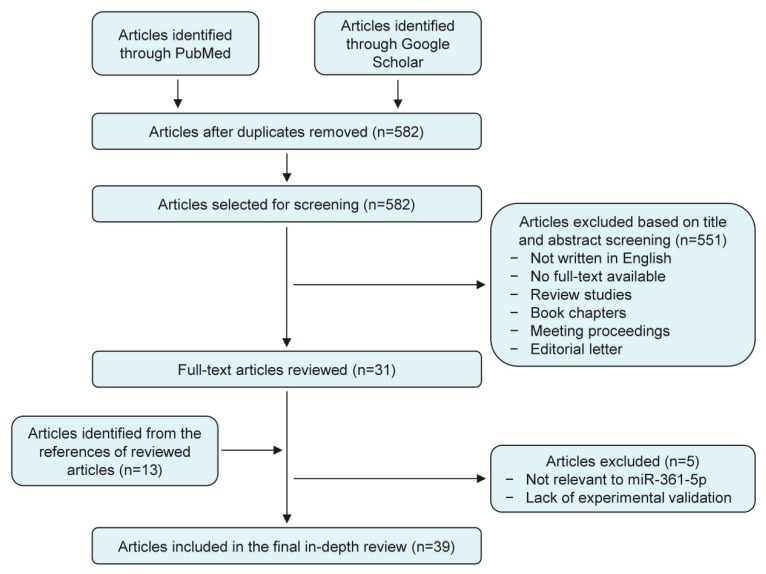
Summary of literature search, screening and selection.

PubMed and Google Scholar were used to search for articles published up to April 2019 using the following keywords: miR-361-5p, microRNA-361-5p, tumor, cancer, and carcinoma. All recognized studies were assessed for relevance by two authors by checking the title and abstract. All irrelevant articles, studies without access to the full text of the publication, case reports, letters, expert opinions, meeting proceedings, review articles, non-English articles, and articles whose methods do not contain biomedical experimental validation were excluded. After this, the full text of any selected article was reviewed independently by two authors. A weakness of our study relates to the lack of access to some relevant research works that may contain information on miR-361 and its target genes. We also searched the reference lists of the reviewed articles to identify additional relevant articles. A flow diagram of the study selection process is shown in Figure 1.

## 3. Dysregulation of miR-361 in Tumor

Previous studies of solid tumors showed that miR-361 is frequently downregulated in various tumor tissues, including cutaneous squamous cell carcinoma [17], osteosarcoma [18], breast cancer [19,20,21,22], glioma [23,24], papillary thyroid carcinoma [25], lung cancer [26,27,28,29], gastric cancer [30,31,32], hepatocellular carcinoma [33], colorectal cancer [32], ovarian cancer [34], endometrial cancer [10], cervical cancer [35], and prostate cancer [36,37,38]. However, increased expression of miR-361 was detected in acute myeloid leukemia [39], indicating the possibility that miR-361 dysregulation might be required to impair differentiation programs in leukemia, and miR-361 may regulate the expression of the hematopoietic differentiation-specific genes, which have a weak importance in solid tumors. Several works demonstrated that low levels of miR-361 were associated with shorter overall survival in patients with breast cancer [20,21], gastric cancer [32], and colorectal cancer [32].

## 4. Mechanisms of miR-361 Regulation in Tumor

**Figure 2:**
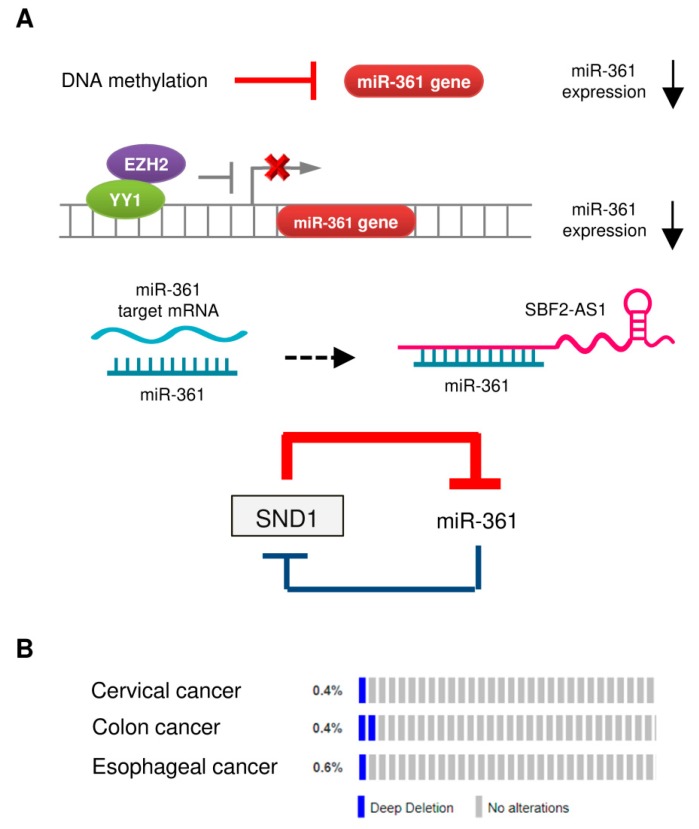
Mechanisms of miR-361 dysregulation in tumors. (**A**) Reported mechanisms responsible for miR-361 downregulation in tumors. (**B**) OncoPrint of cBioPortal showing the genetic alterations of *miR-361* (deep deletion) in tumor samples obtained from The Cancer Genome Atlas (TCGA)-cervical cancer, TCGA-colon cancer, and TCGA-esophageal cancer datasets. Each bar indicates the individual cases and % on the left indicates the percentage of cases altered in the human *miR-361* gene.

Large-scale profiling studies have revealed that dysregulation of miRNA is a common event during cancer carcinogenesis and metastasis [11,12]. The molecular mechanisms regulating miRNA expression include genomic amplification or deletion of miRNA genes, abnormal transcriptional control of miRNAs, epigenetic silencing, and defects in miRNA biogenesis and processing machinery [11,40]. The downregulation of miR-361 in tumor tissues could be caused by several mechanisms (Figure 2A).

### 4.1. DNA Hypermethylation

Hypermethylation of tumor suppressor gene promoter regions leads to silencing of those genes. A previous study reported that the expression of miR-361 in hepatocellular carcinoma cell lines was restored upon treatment with an inhibitor of DNA methylation 5-azacytidine (5-AZA) [33]. Similar results were obtained from another study, where 5-AZA treatment significantly upregulated miR-361 expression in endometrial cancer cells [10].

### 4.2. Transcriptional Control of miR-361 Expression

We previously reported that enhancer of zeste homolog 2 (EZH2), which represses gene expression by methylation of histone H3 on lysine 27, acted as a co-suppressor of transcription factor YY1 to epigenetically suppress the transcription of miR-361 [10]. The use of GSK343 (a specific EZH2 inhibitor) was found to increase the levels of miR-361 in endometrial cancer cells [10].

### 4.3. Long Non-Coding RNA (lncRNA) SBF2-AS1 Acts as a Sponge for miR-361 

LncRNAs are non-protein coding transcripts longer than 200 nucleotides that could serve as miRNA sponges to inhibit the interaction between miRNA-target mRNAs or suppressing the levels of miRNAs [41]. For instance, in mouse cardiomyocytes, mitochondrial dynamic related lncRNA (MDRL) directly binds to miR-361 and acts as its sponge to promote the processing of pri-miR-484 [42]. Another lncRNA, Maternally Expressed Gene 3 (MEG3), was shown to facilitate cardiac hypertrophy by sponging miR-361 [43]. In cervical cancer cells, lncRNA SBF2-AS1 (SBF2 Antisense RNA 1) was shown to function as an endogenous RNA sponge that interacted with miR-361 and suppressed its expression [35].

### 4.4. The SND1/miR-361 Feedback Loop Controls miR-361 Expression 

miR-361 directly targeted SND1 (Staphylococcal nuclease and tudor domain containing 1), and SND1 conversely suppressed the expression of miR-361 by binding to pre-miR-361, thus creating a double-negative feedback loop, in which miR-361 and SND1 repress expression of each other in gastric and colon cancer cells [24].

### 4.5. Deletion of the Human miR-361 Gene 

The reduced miRNA expression in tumor cells could arise from copy-number alterations and chromosomal aberrations (such as amplification, deletion, or translocation) [11]. However, whether genomic alterations of the human miR-361 gene can lead to decreased expression of miR-361 in cancer is poorly understood. We investigated copy-number alterations and nucleotide changes of the miR-361 gene in human cervical, colon and esophageal cancer samples from the cBioPortal database. As shown in Figure 2B, gene deletion is the most frequent alteration type in these cancers, supporting the notion that the loss of miR-361 expression plays an important role in the development of colon and cervical cancers [32,35].

## 5. The Impact of miR-361 on the Aggressive Properties of Tumor Cells and Tumor Microenvironment Remodeling

**Figure 3:**
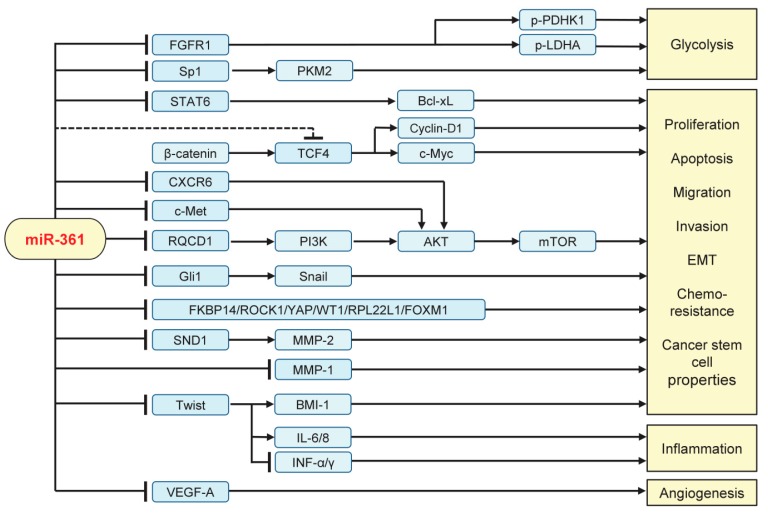
Validated targets and signaling pathways regulated by miR-361 in human tumor cells. FGFR1: Fibroblast growth factor receptor 1; Sp1: Transcription factor; STAT6: Signal transducer and activator of transcription 6; PKM2: pyruvate kinase M2; PDHK1: pyruvate dehydrogenase kinase 1; LDHA: lactate dehydrogenase A; TCF4: Transcription factor 4; CXCR6: C-X-C chemokine receptor type 6; RQCD1: CCR4-NOT transcription complex subunit 9; PI3K: phosphoinositide 3-kinase; mTOR: mammalian target of rapamycin; SND1: Staphylococcal nuclease and tudor domain containing 1; MMP: Matrix metallopeptidase; IL: interleukin; INF-α/γ: interferon α/γ; VEGF-A: vascular endothelial growth factor A.

MiR-361 has been shown to act as a novel tumor suppressor that represses a large number of downstream target transcripts implicated in cellular proliferation, glycolysis, migration, invasion, EMT, chemoresistance, cancer stemness, angiogenesis, and inflammation (Figure 3).

### 5.1. Inhibiting Tumor Growth, Invasion, EMT, Metastasis, and Glycolysis

Restoration of miR-361 expression by transfection with miR-361 mimics inhibited the proliferation of osteosarcoma, breast cancer, thyroid papillary carcinoma, lung cancer, gastric cancer, colorectal cancer, hepatocellular carcinoma, cervical cancer, and prostate cancer cells [18,19,25,26,27,28,29,30,31,32,33,35,36]. Moreover, over-expression of miR-361 attenuated cell migration and invasion in endometrial cancer, breast cancer, glioma, papillary thyroid carcinoma, lung cancer, gastric cancer, ovarian cancer, and prostate cancer [10,19,23,24,25,26,28,30,32,34,36]. Conversely, knocking down miR-361 expression using anti-miR-361 inhibitor promoted cell migration and invasion in endometrial cancer, breast cancer, glioma, papillary thyroid carcinoma, lung cancer, gastric cancer, and ovarian cancer [10,19,22,23,24,25,26,28,30,32,34,36]. Mechanically, miR-361 impairs tumor cell proliferation, migration, and invasion by directly targeting and downregulating the expression of FKBP14 [18], MMP-1 [19], SND1 [24], ROCK1 [25], YAP [27], WT1 [28], RPL22L1 [34] and STAT6 [29,37].

The phosphoinositide 3-kinase (PI3K)/AKT pathway is activated in a wide range of cancers, and associated with cell growth, proliferation, survival, motility, tumor progression and resistance to cancer therapies. CCR4-NOT transcription complex subunit 9 (CNOT9/RQCD1) has been identified as a key activator of the PI3K/AKT pathway [44], and upregulation of miR-361 in breast cancer cells can suppress its direct target RQCD1, leading to the downregulation of PI3K, AKT, and MMP-9 [22]. The C-X-C motif chemokine receptor 6 (CXCR6), when bound with its ligand CXCL16, induced the activation of the PI3K/AKT signaling in cancer cells [45]. A previous study demonstrated that miR-361 inhibited the proliferation of hepatocellular carcinoma cells by directly targeting CXCR6 [33]. These results provided examples of miR-361-mediated repression of the PI3K/AKT signaling at different levels and illustrated the importance of miR-361 regulation in carcinogenesis and tumor progression. EMT encompasses a series of phenotypic and biochemical changes that enable epithelial cells to acquire a mesenchymal cell phenotype, which includes enhanced migration, invasion, metastasis, CSC-like features, resistance to conventional chemotherapy, radiotherapy, and small-molecule drugs [46,47,48].

EMT is mediated by a core set of key transcription factors, including Twist, Zinc finger E-box binding homeobox 1 (ZEB1), ZEB2, Snail and Slug, and the expression of these transcription factors are finely mediated at the transcriptional, translational, and post-translational levels [46,47,48].

In accordance with its reported anti-tumor functions, ectopic expression of miR-361 was found to cause dramatic suppression of EMT process in various cancer cells. For example, experiments show that enforced overexpression of miR-361 greatly suppressed EMT, invasion and metastasis in many tumors, including endometrial cancer, glioma, lung cancer, gastric cancer, colorectal cancer, ovarian cancer, and prostate cancer [10,23,26,32,34,36]. By inhibiting the expression of Twist, miR-361 played a crucial role in suppressing EMT characteristics and cancer stem cell (CSC)-like properties of endometrial cancer cells [10].

In addition to targeting EMT-promoting transcription factors directly, miR-361 also modulated the expression of key mediators of the EMT program. For example, the loss of miR-361 expression activated Gli1 and its downstream effector Snail to promote EMT and prostate cancer cell invasion [36]. In ovarian cancer cells, miR-361 targeted and reduced the levels of RPL22L1 and another target gene *c-Met* [34], which could serve as an upstream stimulator of the PI3K/AKT signaling and EMT-associated signaling pathways [49]. Additionally, miR-361 attenuated EMT and chemoresistance in cancer cells by suppressing the expression of FOXM1 [26,31], an oncogenic transcription factor required for EMT and metastasis [50].

Activation of the Wingless-type MMTV integration site family (Wnt)/β-catenin signaling in cancer cells is responsible for EMT induction, metastasis, CSC self-renewal, increased resistance to chemotherapy or radiotherapy and immunosuppression [51]. Although the introduction of miR-361 into gastric cancer cells downregulated the expression of Wnt/β-catenin pathway-related proteins (TCF4, Cyclin-D1 and c-Myc) [30], it remains unknown whether these genes are direct targets of miR-361.

Cancer cells are known to consume more glucose to produce lactate by glycolysis rather than oxidative phosphorylation, even in oxygen-rich conditions [52]. Recent data suggested that miR-361 directly targeted the 3′-UTR of *FGFR1*, which promotes glycolysis through activation of two critical glycolytic enzymes lactate dehydrogenase A (LDHA) and pyruvate dehydrogenase kinase 1 (PDK1/PDHK1), thereby suppressing glucose consumption and lactate production of breast cancer cells [19]. The glycolytic enzyme pyruvate kinase M2 (PKM2) is often highly expressed in cancer cells but is present at a very low level in normal cells [52]. PKM2 catalyzes the rate-limiting ATP-generating step of glycolysis, controlling the conversion of phosphoenolpyruvate and ADP to pyruvate and ATP, respectively [52]. Transfection of miR-361 mimic was shown to inhibit glucose metabolism by targeting Sp1 and subsequently downregulating the expression of PKM2 in prostate cancer [38]. The role of miR-361 in the regulation of glucose metabolism in human cancers has not yet been fully investigated.

### 5.2. Suppressing Angiogenesis and Inflammation

Accumulated evidence showed that miRNAs participate in the remodeling of tumor microenvironments through several mechanisms, including the regulation of the expression of cell membrane proteins, secretion of cytokines, as well as transmission of mature miRNAs between different cell types via exosomes [53,54]. It has become apparent that miR-361 is able to regulate cancer progression through modulating tumor microenvironments (Figure 3).

Angiogenesis, an important hallmark of cancer, plays an essential role in providing tumor cells with oxygen and nutrients. Some miRNAs modulate the expression of regulatory molecules driving angiogenesis, including vascular endothelial growth factors (such as vascular endothelial growth factor A (VEGF-A)), cytokines, metalloproteinases, and growth factors [55]. MiR-361 targeted the 3′-UTR of *VEGF-A* to repress its expression in skin squamous cell carcinoma [17]. Moreover, overexpression of miR-361 was shown to indirectly reduce the expression of VEGF-A through inhibiting the Wnt/β-catenin pathway in gastric cancer cells [30]. Consistent with these data, transient transfection with miR-361 mimic significantly downregulated the expression of VEGF-A, whereas the silencing of miR-361 with miRNA inhibitor enhanced the levels of VEGF-A in endometrial cancer cells [10]. Collectively, these results suggest that reduced levels of miR-361 could be an important driving mechanism for the formation of a pro-angiogenic tumor microenvironment.

Numerous studies have indicated that chronic inflammation actively promotes tumor initiation, progression, and metastasis via multiple mechanisms, including generation of an immunosuppressive tumor microenvironment [56]. Tumor cells undergoing EMT could modulate the surrounding microenvironment via enhanced secretion of inflammatory cytokines (including IL-6 and IL-8) [57,58]. We reported that miR-361 could downregulate the mRNA expression of IL-6 and IL-8 in endometrial cancer cells through targeting Twist [10]. Additionally, the activation of signal transducer and activator of transcription (STAT) family members (for example STAT6) is closely linked to tumor-promoting inflammation and the suppression of anti-tumor immunity in multiple cancer tissues [59,60]. MiR-361 was shown to directly inhibit the expression of STAT6 by binding to its 3′-UTR region [29,37]. These data support a novel function of miR-361 in exerting anti-angiogenesis and anti-inflammatory effects, at least by regulating the EMT-associated signaling and the production of pro-inflammatory cytokines.

## 6. Diagnostic and Prognostic Value of miR-361 in Tumor

Early studies showed that miRNA expression signatures can be useful in distinguishing cancer tissues from normal tissues, categorizing cancer subtypes, and predicting the progression, prognosis, and treatment response in many cancer types [61,62,63,64,65]. miRNAs are more stable than mRNA in the peripheral blood, serum, and formalin-fixed tissues [61,62] and often exhibit tumor-specific and tissue-specific expression profiles, making them attractive candidates for diagnostic and prognostic applications.

More specifically, downregulation of miR-361 was implicated in the progression of many tumor types, including breast cancer [19,21], glioma [24], papillary thyroid carcinoma [25], and lung cancer [66]. Lower miR-361 levels in patients with breast cancer [19,21], colon cancer [32], and lung cancer [66] were associated with shorter overall survival, suggesting that reduced miR-361 expression serves as a potential biomarker that predicts poor clinical outcomes in cancer patients.

Circulating miRNAs escape degradation by residing within microvesicles, exosomes, and apoptotic bodies, and dysregulated miRNAs have been detected in the blood, plasma, and serum of cancer patients [67]. The levels of circulating miRNAs (such as let-7, miR-155 and miR-195) were able to distinguish those patients with breast cancer from healthy controls [68]. Another study showed that several circulating miRNAs were detected in stage I/II breast cancer patients’ plasma at a significantly higher level compared to healthy controls, suggesting that these miRNAs might be used for early cancer diagnosis [69]. Changes in circulating miRNA expression were linked to lymph node metastasis in breast cancer patients [70]. Furthermore, the diagnostic value of a panel of cancer-associated miRNAs was verified in patients with various cancer types [71]. To date, only two studies described miR-361 signatures in plasma of patients with cancers [39,72]. Quantitative PCR analysis of a group of miRNAs in acute myeloid leukemia (AML) indicated that miR-361 was significantly increased in plasma of newly diagnosed AML patients at diagnosis compared to healthy controls and decreased after chemotherapy [39]. In addition, deep sequencing of circulating miRNAs in plasma of lung cancer patients demonstrated that the levels of miR-361 were upregulated in cancer patients compared with healthy controls, and were relatively higher in patients with adenocarcinoma than in squamous cell carcinoma [72], suggesting that circulating miR-361 may be used for the differentiation of different cancer subtypes.

## 7. Treating Cancer with miR-361 Replacement Therapy

Although many miRNA-based therapeutics are processed in the preclinical stage, only one miRNA therapeutic, the compound SPC3649 (miravirsen, an inhibitor of miR-122) has undergone successful phase II clinical trials for the treatment of hepatitis C virus. Therapeutically restoring the expression of tumor suppressor miRNAs using synthetic miRNA mimics or miRNA expression plasmids has been developed for the clinical modulation of miRNAs [73]. Numerous studies showed that reintroduction of miR-361 exhibited significant anti-tumor activities in experimental xenograft models of breast cancer, thyroid papillary carcinoma, lung cancer, gastric cancer, colorectal cancer, hepatocellular and prostate cancer [19,25,26,30,32,33,37], highlighting its potential as a therapeutic target for treatment of these cancers. In endometrial cancer cells, we identified that EZH2 was a key upstream suppressor of miR-361, and showed that EZH2 blockade using GSK343, a specific EZH2 inhibitor that showed effective anti-cancer effects and minimal toxicity against normal cells, led to the reactivation of miR-361 and the suppression of endometrial cancer progression [10]. These findings provided an insightful cancer therapeutic strategy to indirectly restore miR-361 function via targeting EZH2.

## 8. Future Perspectives

Although it is becoming clear that miR-361 exerts a tumor-suppressive function in most solid tumors via reducing cancer aggressiveness and producing a suppressive tumor microenvironment, the multifaceted biological roles of miR-361 are yet to be fully characterized.

**Figure 4:**
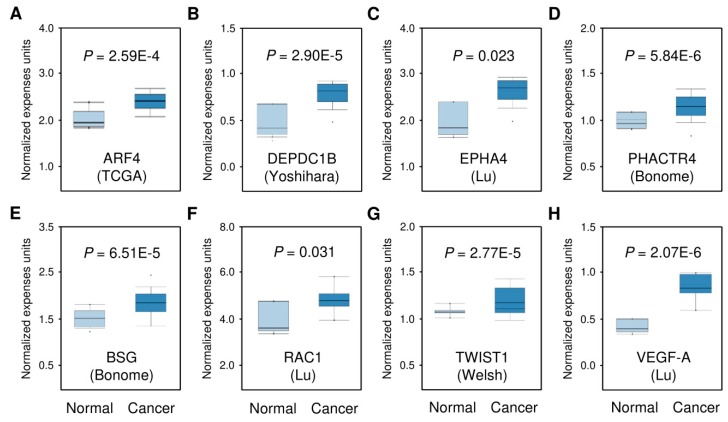
Oncomine analysis indicates higher expression of the predicted miR-361 targets in ovarian cancer tissues. The box plots revealed the expression levels of *ARF4* (**A**, The Cancer Genome Atlas (TCGA)), *DEPDC1B* (**B**, Yoshihara), *EPHA4* (**C**, Lu), *PHACTR4* (**D**, Bonome), *BSG* (**E**, Bonome), *RAC1* (**F**, Lu), *Twist* (**G**, Welsh) and *VEGF-A* (**H**, Lu) in ovarian cancer tissues with respect to normal tissues. *P*-values were calculated using the Oncomine database through two-sided Student’s *t*-test. A value of *p* < 0.05 was considered as statistically significant.

Using the TargetScan, miRDB, and miRSystem online analysis tools, we explored the potential target genes of miR-361. All these tools identified over 200 overlapping target genes for miR-361. However, only a small proportion of these miR-361 target genes (around 8%) have been experimentally validated in tumor cells (Figure 3). As shown in Figure 4, we identified six unreported miR-361 targets (*ARF4*, *DEPDC1*, *EPHA4*, *PHACTR4*, and *BSG*) and two previously reported miR-361 targets (*Twist* and *VEGF-A*) [10,17]. We investigated the expression of these genes in human ovarian cancer tissues and normal tissues using the Oncomine database (https://www.oncomine.org/resource/login.html). The expression levels of these genes were significantly increased in ovarian cancer tissues (Figure 4), indicating that these genes might be important components of miR-361-mediated gene networks.

Despite the recent progress that has been made towards the identification of the molecular mechanisms causing dysregulation of miR-361, there are currently many unclear points. Given that lncRNAs interact with miRNAs to form the intertwined and regulatory networks that control cancer development and progression [74], detailed analysis of the interactions between lncRNAs and miR-361 may partly explain the frequent downregulation of miR-361 observed in cancers. With the perspective of therapeutic miR-361 restoration, the existence of upstream suppressors (such as EZH2) should be taken into consideration.

The most common types of genetic variations in the human genome are single nucleotide polymorphisms (SNPs), which are the results of point mutations that produce single base-pair differences among chromosome sequences [75]. SNPs are located in different regions of genes (such as promoters, exons, introns, 5′-UTRs, and 3′-UTRs) and alter gene expression through complex mechanisms [76]. The occurrence of SNPs may affect cancer susceptibility and represent genetic markers for cancer risk [77,78]. The function of miRNAs may be influenced through SNPs in their own sequences and in their target gene sequences [79]. Some SNPs were shown to interfere with the function of certain miRNAs and affect the expression of the miRNA targets [80]. A study confirmed that a functional SNP in *CD80* 3’-UTR disrupted the inhibitory effect of miR-361 on CD80 expression in gastric cancer cells [81]. Further characterization of SNPs in the miR-361 gene and its potential targets in cancer cells would shed light on the molecular mechanisms responsible for miR-361-mediated carcinogenesis and metastasis.

## 9. Conclusions

Emerging works on miR-361 demonstrated its importance in controlling multiple malignant features of tumor cells and regulating critical aspects of the tumor microenvironment. MiR-361 has great potential to be used as a promising diagnostic, prognostic, and predictive biomarker for cancers and has therapeutic potential to improve cancer treatment. Additional works will continue to elucidate how miR-361 exerts significant effects on tumor progression and will offer crucial therapeutic opportunities for cancer patients.
